# Ginsenoside Rb1 Prevents H_2_O_2_-Induced HUVEC Senescence by Stimulating Sirtuin-1 Pathway

**DOI:** 10.1371/journal.pone.0112699

**Published:** 2014-11-11

**Authors:** Zhiming Song, Yong Liu, Baoshun Hao, Shujie Yu, Hui Zhang, Dinghui Liu, Bin Zhou, Lin Wu, Min Wang, Zhaojun Xiong, Chaodong Wu, Jieming Zhu, Xiaoxian Qian

**Affiliations:** 1 Department of Cardiology, The Third Affiliated Hospital of Sun Yat-sen University, Guangzhou, Guangdong, China; 2 Department of Ultrasonography, The Third Affiliated Hospital of Sun Yat-sen University, Guangzhou, Guangdong, China; 3 Department of Nutrition and Food Science, Texas A&M University, College Station, TX, United States of America; 4 Institute Integrated Traditional Chinese and Western Medicine, Sun Yat-sen University, Guangzhou, Guangdong, China; Uniformed Services University, United States of America

## Abstract

**Purposes:**

We have previously reported that Ginsenoside Rb1 may effectively prevent HUVECs from senescence, however, the detailed mechanism has not demonstrated up to now. Recent studies have shown that sirtuin-1 (Sirt1) plays an important role in the development of endothelial senescence. The purpose of this study was to explore whether Sirt1 is involved in the action of Ginsenoside Rb1 regarding protection against H_2_O_2_-induced HUVEC Senescence.

**Methods and Results:**

Senescence induced by hydrogen peroxide (H_2_O_2_) in human umbilical vein endothelial cells (HUVECs) was examined by analyzing plasminogen activator inhibitor-1 (PAI-1) expression, cell morphology, and senescence-associated beta-galactosidase (SA-β-gal) activity. The results revealed that 42% of control-treated HUVECs were SA-β-gal positive after treatment by 60 µmol/L H_2_O_2_, however, this particular effect of H_2_O_2_ was decreased more than 2-fold (19%) in the HUVECs when pretreated with Rb1 (20 µmol/L) for 30 min. Additionally, Rb1 decreased eNOS acetylation, as well as promoted more NO production that was accompanied by an increase in Sirt1 expression. Furthermore, upon knocking down Sirt1, the effect of Rb1 on HUVEC senescence was blunted.

**Conclusions:**

The present study indicated that Ginsenoside Rb1 acts through stimulating Sirt1 in order to protect against endothelial senescence and dysfunction. As such, Sirt1 appears to be of particular importance in maintaining endothelial functions and delaying vascular aging.

## Introduction

Cardiovascular diseases (CVD) are the leading cause of death and disability in the world, especially among an aged population. Because CVD mortality rates increase with age in the later years of life, the aging process is recognized as the main risk factor for the development of CVD[1].

Aging involves a natural decline in the chances of survival that all species experience with increasing age [2]. Biological aging is termed senescence. The process involves numerous changes to the molecular and cellular structures disrupting metabolism, eventually leading to deterioration or death [3]. Senescence is classified as organismal senescence and cellular senescence. Because organismal senescence is composed of cellular senescence, increased consideration has been given to cellular senescence.

Cellular senescence was first described by Hayflick and his colleagues in 1961 [4], when they made the observation that normal human fibroblasts would enter into an irreversible state of growth cessation after several continuous passages. This phenomenon was called replicative senescence. Thereafter, researchers found many stressors that are able to induce senescence that is known as stress-induced premature senescence (SIPS). Among many of the stressors, hydrogen peroxide (H_2_O_2_) is a better candidate for inducing senescence, because an H_2_O_2_-induced process could mimic the oxidative environment that occurs in the aging population with high efficiency [5].

The vascular endothelium is a thin layer of cells lining the innermost surface of blood vessels that acts as a semi-selective barrier, preventing lipid infiltration. Its dysfunction is a progressive and multifactorial phenomenon in the elderly. Also, it is accepted that endothelial dysfunction is an independent risk factor for the development of atherosclerosis, which is more obvious during aging [6], [7]. The close relationship between aging and endothelial dysfunction points to a critical need to find ways to protect against endothelial senescence.

Ginsenosides are a class of steroid glycosides found exclusively in the plant genus, panax (ginseng). Much evidence has demonstrated that ginseng generates beneficial effects on health. In the United States of America, ginseng ranks second and fifth among the 10 most common natural products used by adults in 2002 and 2007, respectively [8]. Ginsenosides are divided into two groups: Rb1 group and Rg1 group. Ginsenoside Rb1 (Rb1) is one of the most abundant ginsengs and also attracts much attention [9]. Rb1 affects the reproductive system and embryo development. Recently, an increasing amount of evidence has demonstrated that Rb1 could increase endothelial nitric oxide synthase (eNOS) and reverse H_2_O_2_- or homocysteine-induced endothelial dysfunction in vivo and in vitro [10]–[12].

Sirtuin-1 (Sirt1) is an NAD^+^-dependent lysine deacetylase, and it has been recognized to play important roles in cell aging, organism longevity, and stress responses [13]–[15]. Sirt1 increased eNOS-derived nitric oxide (NO) through the deacetylation of eNOS [16], [17]. H_2_O_2_ treatment reduces its expression in human lung epithelial cells and causes a dose-dependent reduction in human endothelial cells [18], [19].

Our previous studies have shown that Rb1 prevented HUVEC senescence through modulating redox status [11]. However, it is unknown whether Sirt1 is involved in Rb1 prevention of H_2_O_2_-induced HUVEC senescence. The present study provides evidence to support the novel role of Sirt1 in the prevention of the Rb1 effects on HUVEC senescence.

## Materials and Methods

### Cell culture

Primary HUVECs were isolated from the umbilical cord of a newborn as described previously [20], and cells in passages 2–4 were used in this study. Cells were grown in an M199 medium (Hyclone, Thermo-Fisher, MA) containing 20% fetal bovine serum (Gibco, Grand Island, NY), a 20% serum-free medium (Gibco), 60 µg/mL of endothelial cell growth supplement (BD, San Diego, CA), and 100 U/mL of penicillin with 100 µg/mL of streptomycin (Gibco). The procedure for HUVEC isolation was approved by the Research Committee at the Third Affiliated Hospital of Sun Yat-sen University. The parturients enrolled in this protocol were negative for human immunodeficiency virus, hepatitis B virus and signed the written informed consent to providing their umbilical cords.

### Induction of senescence in primary HUVECs

To induce premature senescence, the isolated HUVECs were treated with different concentrations of H_2_O_2_ (Sigma, St. Louis, MO) for 1 hour and then culture for another 24 hours. In the experiments, for testing the effect of Rb1 (Victory, Sichuan, China) on senescence, the cells were pretreated with 20 µmol/L of Rb1 for 30 min before the addition of H_2_O_2_.

### Flow cytometry

An Annexin V-FITC apoptosis detection kit (Keygen, Nanjing, China) was used to detect the apoptosis of HUVECs. Flow cytometry was performed as per the manufacturer’s instructions. Briefly, cells were collected with 5% trypsin without EDTA and washed with phosphate buffer solution (PBS). After re-suspension in 500 µL of a binding buffer, the cells were added to 5 µL of Annexin V-FITC and 5 µL of propidium iodide and then were completely mixed. After incubation for an additional 10 minutes in the dark, Annexin V-FITC binding was analyzed in the labeled cells by using flow cytometry (Ex = 488 nm; Em = 530 nm).

### Cell viability

The cells were seeded in a 96-well plate, treated with H_2_O_2_ for 1 h, and incubated for an additional 24 hours. Cell viability was evaluated by using the MTT assay. Briefly, 20 µL of 5 mg/mL MTT (Sigma) was added to the cells in each well and incubated for 4 hours at 37°C. After carefully removing the medium and adding 150 µL of DMSO to each well, the cell plates were covered with tinfoil, agitated on a shaker for 15 minutes, and then subjected to a plate reader to read absorbance at 490 nm.

### SA-β-gal staining

Senescence staining was performed according to a prior protocol [21]. Briefly, the cells were rinsed twice with a 1× PBS for 30 seconds and fixed with 2% formaldehyde plus 1% glutaraldehyde for 10 minutes at room temperature. The cells were then washed twice as described above and incubated with a staining solution (40 mmol/L of citric acid/sodium phosphate buffer, 5 mmol/L of K_4_[Fe(CN)_6_]3H_2_O, and 5 mmol/L of K_3_[Fe(CN)_6_], 150 mmol/L of sodium chloride, 2 mmol/L of magnesium chloride, and 1 mg/mL of X-gal) overnight at 37°C without CO_2_. The proportion of SA-β-gal–positive cells was calculated by counting 400 cells in each group.

### Immunoblotting and immunoprecipitation

The cells were washed with PBS and lysed on ice for 15 minutes in an RIPA buffer (Keygentech, Nanjing, China). Equal amounts of protein (30 µg) were separated by SDS-page gel electrophoresis and transferred to nitrocellulose membranes. After blocking, the membranes were incubated at 4°C overnight with the following antibodies: Sirt1 (1∶1000), phosphorylated Sirt1 (Ser47, 1∶2000), acetylated-lysine (1∶1000) and eNOS (1∶1000) are purchased from Cell Signaling Technologies (Danvers, MA); PAI-1 (1∶10000, Santa Cruz, CA) and GAPDH (1∶20000, Proteintech, IL). After washing and incubating with an HRP-conjugated secondary antibody (Boster, Wuhan, China), the membrane was visualized on an x-ray film by using an ECL reagent. Immunoprecipitation was performed according to the manufacturer’s instructions. Briefly, a total of 100 µg of protein was transferred to a 1.5-mL tube and incubated with 0.3 µg of eNOS antibody on a rocker at 4°C for 4 hours. After adding 20 µL of a Protein A/G PLUS agarose (Santa Cruz, CA), the mixture was incubated at 4°C on a rocker overnight. After centrifugation at 2500 rpm for 5 minutes at 4°C, the immunoprecipitates were collected, washed with PBS 4 times, and subjected to immunoblotting as described above.

### Quantitative real-time polymerase chain reaction

The total RNA was extracted from the HUVECs by using RNAiso Plus (Takara, Dalian, China). Complementary DNA was generated with a revertaid first-strand cDNA synthesis kit (Thermo Scientific, MA). A DyNAmo ColorFlash SYBR Green qPCR Kit (Thermo Scientific, MA) was then used for a real-time polymerase chain reaction (PCR) reaction examination. The following primers were used: Sirt1(GenBank No. NM_012238.4) forward (F), 5′-TGT GGT AGA GCT TGC ATT GAT CTT-3′ and reverse (R), 5′-GGC CTG TTG CTC TCC TCA TT-3′; PAI-1(GenBank No. NM_001165413) F, 5′-TGC TGG TGA ATG CCC TCT ACT-3′ and R, 5′-CGG TCA TTC CCA GGT TCT CTA-3′; and β-actin(GenBank No. NM_001101.3) F, 5′-AGC GGG AAA TCG TGC GTG AC -3′ and R, 5′-TCC ATG CCC AGG AAG GAA GG-3′. The relative expression of the target gene was normalized against β-actin and shown as 2^−[Ct(target)−Ct(β−actin)]^.

### RNA interference

A small interfering RNA (siRNA) of Sirt1, as well as a negative control, was synthesized by Genepharma (Shanghai, China). Proliferating cells were transfected with 100 nmol/L of siRNA for Sirt1 (5′-GGU CAA GGG AUG GUA UUU ATT-3′ and 5′-UAA AUA CCA UCC CUU GAC CTT-3′) or a scrambled control siRNA (5′-UUC UCC GAA CGU GUC ACG UTT-3′ and 5′-ACG UGA CAC GUU CGG AGA ATT-3′) by using Lipofectamine RNAiMAX (Life Technologies, Carlsbad, CA). After a 6-hour siRNA transfection, the medium was changed, and the cells were treated with 60 µmol/L of H_2_O_2_ or continued to grow for the indicated hours.

### Measurement of NO concentration

The NO levels in the cell culture supernatant were measured by using an NO assay kit (Nanjing Jiancheng Institute of Biological Engineering, Nanjing, China). Briefly, a total of 100 µL of sample and standard (S) or distilled water (B) was added to a 1.5-mL tube. After adding 200 µL of reagents 1 and 2, the tubes were well mixed and incubated at 37°C for 1 hour. After adding 200 µL of reagent 3 and 100 µL of reagent 4 to each tube, the mixtures were thoroughly mixed for 30 seconds and incubated at room temperature for 40 minutes. After incubation, the tubes were centrifuged at 3500 rpm for 10 minutes. A total of 400 µL of the supernatants was transferred to new tubes and added with 600 µL of the color development reagent 5 into each tube. After mixing well, the tubes were incubated for 10 minutes. A total of 200 µL of each sample and the standard or blank solution were transferred to the 96-well plate in order to read the absorbance at 550 nm. The NO levels were calculated as follows:

NO concentration = [OD(sample)–OD(B)]/[OD(S)–OD(B)]×100 µmol/L.

### Statistical analysis

Numeric data are expressed as mean ± SEM. The difference between groups was analyzed by a one-way analysis of variance or an independent sample *t*-test. Results were considered statistically different if the p-value was less than 0.05.

## Results

### HUVEC senescence that was induced by treatment with H_2_O_2_

**Figure 1:**
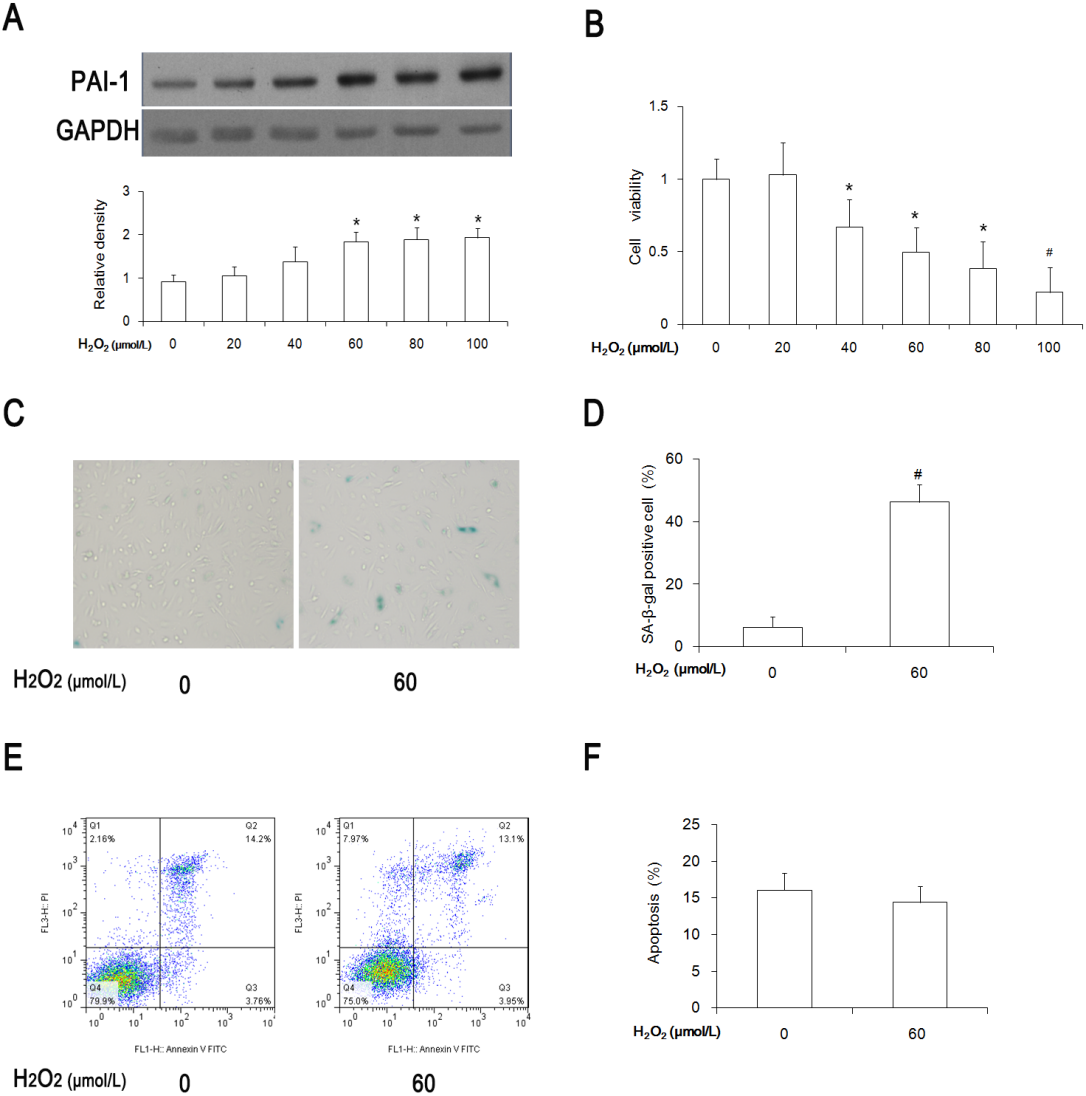
Premature senescence was induced 24 hours after a 1-hour H_2_O_2_ treatment. (A) The PAI-1 protein levels with different dosages in the H_2_O_2_-treated groups. (B) Cell viability detected with an MTT assay. (C) SA-β-gal staining was performed in each group. Senescent cells were stained with blue color. (D) The ratio of SA-β-gal positive cells was calculated on 400 cells per group. (E) An analysis of apoptosis by flow cytometry. (F) Statistical analysis of apoptosis in each group. *P<0.05 vs 0 group, n = 3–4.

In order to establish a senescence model, HUVECs were treated with different dosages (0, 20, 40, 60, 80, and 100 µmol/L) of H_2_O_2_ for 1 hour and then cultured for another 24 h. The protein expression of PAI-1, a senescence marker, increased in a dose-dependent manner and reached a significant difference if the cells were treated with 60 µmol/L of H_2_O_2_, as compared with the control group (Figure 1A). At the same time, cell viability dramatically decreased with an increased H_2_O_2_ concentration (Figure 1B).

Considering the PAI-1 protein expression and cell viability, 60 µmol/L of H_2_O_2_ was chosen to induce senescence. From the SA-β-gal–staining result, HUVEC senescence was effectively induced with 60 µmol/L of an H_2_O_2_ treatment (Figure 1C and 1D).

Previous studies have reported that H_2_O_2_ induced the apoptosis of endothelial cells [22]–[24]. Flow cytometry was performed in order to exclude an apoptotic effect on our results. There was no apparent difference regarding cell apoptosis between the control group and the 60 µmol/L H_2_O_2_ group (16.1% vs 14.4%, Figure 1E and 1F). From the above-mentioned results, 60 µmol/L of H_2_O_2_ was the best concentration to induce senescence, and it was used in our following experiments.

### Effect of Rb1 on senescence and Sirt1 expression

**Figure 2:**
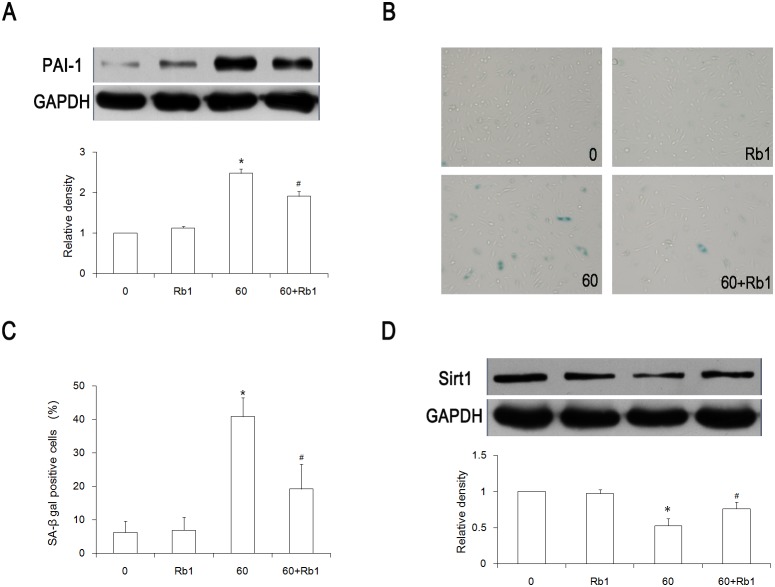
Rb1 inhibited H_2_O_2_-induced senescence and increased Sirt1 expression in the HUVECs. Cells were pretreated with 20 µmol/L of Rb1, treated with or without 60 µmol/L of H_2_O_2_ for 1 hour, and then grown for 24 hours. (A) A Western blot analysis of PAI-1 protein expression. (B) Representative pictures of SA-β-gal staining. (C) The ratio of SA- β-gal–positive cells. (D) Sirt1 expression was analyzed by a Western blot analysis. *P<0.01 vs 0 group; #*P<0.05 vs* 60 group, n = 3. 0, control group; Rb1, 20 µmol/L Rb1-treated group; 60, 60 µmol/L H_2_O_2_–treated group; 60+Rb1, 60 µmol/L H_2_O_2_ plus 20 µmol/L Rb1-treated group.

In our previous study, we found that 20 µmol/L of Rb1 could effectively prevent HUVECs from senescence [11]. As such, we continued to use 20 µmol/L of Rb1 in this study in order to explore its effect. HUVECs were pretreated with 20 µmol/L of Rb1 for 30 min and then treated with 60 µmol/L of H_2_O_2_ for 1 h. After treatment with H_2_O_2_ for 24 hours, the protein levels of PAI-1 and Sirt1 was increased and decreased, respectively, but the effect of H_2_O_2_ on these levels was reversed when pretreated with Rb1(Figure 2A and 2D). The SA-β-gal activity was also detected in order to determine the senescent phenotype of the HUVECs. In the H_2_O_2_ group, about 42% of the cells were SA-β-gal–stain positive, but in the Rb1 plus H_2_O_2_ group, only 19% of the cells were positive (Figure 2B and 2C).

### Inhibition of Sirt1 induced a premature senescent-like phenotype in HUVECs

**Figure 3:**
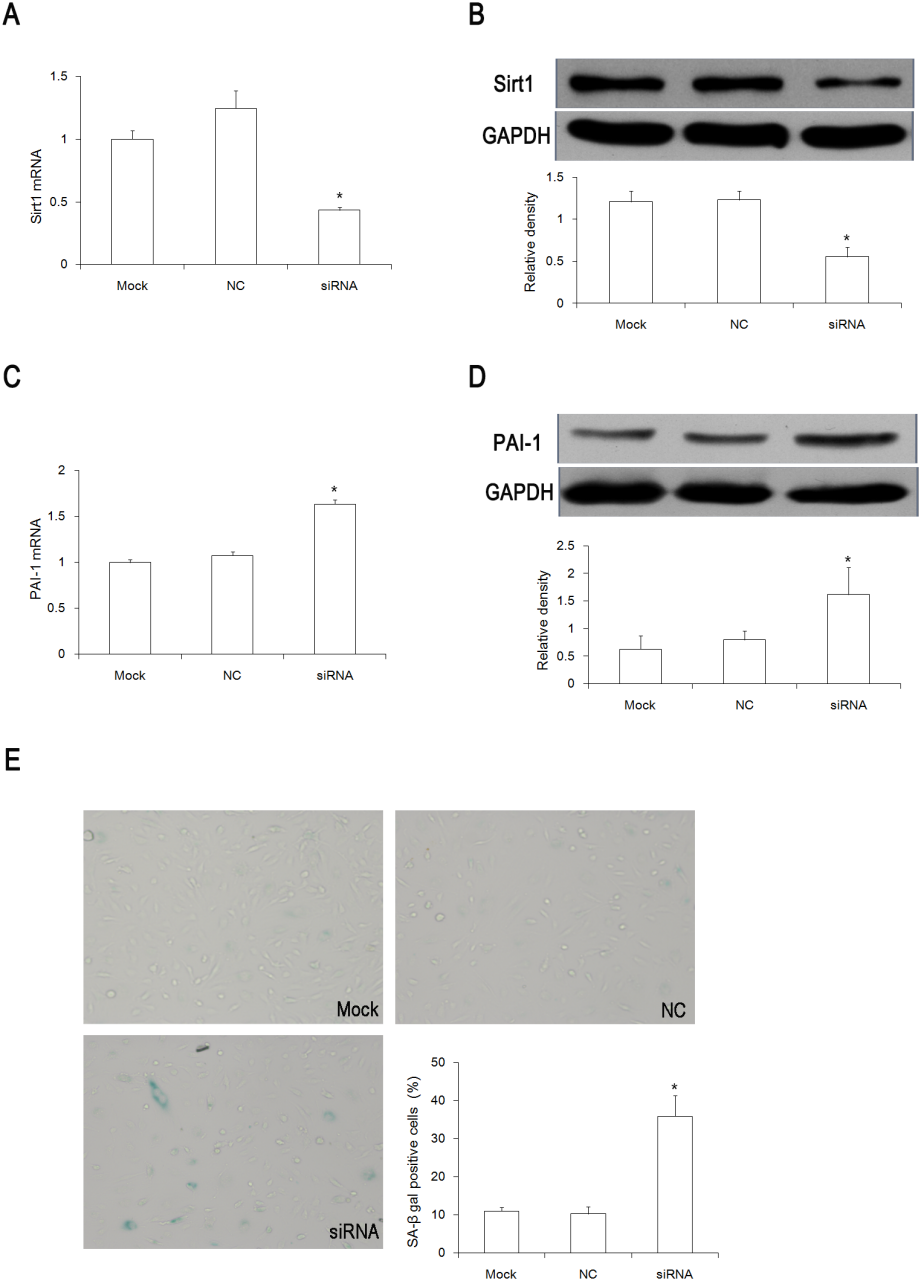
Inhibition of Sirt1 in HUVECs induced premature senescence. (A) and (B) Profiles of *Sirt1* mRNA expression and Sirt1 protein expression 24 hours after transfection with Sirt1 siRNA. (C) and (D) Profiles of *PAI-1* mRNA expression and PAI-1 protein expression 72 hours after transfection with Sirt1 siRNA. (E) SA-β-gal staining was done 72 hours after Sirt1 siRNA transfection. *P<0.05 vs Mock group, n = 3. Mock, transfection reagent only group; NC, negative control siRNA group; siRNA, Sirt1 siRNA group.

With H_2_O_2_-induced senescence, the Sirt1 protein expression was dramatically decreased. In order to fully understand the role of Sirt1 in endothelial cell senescence, Sirt1 was depleted with siRNA. Knockdown of Sirt1 was confirmed by qPCR and Western blotting. Both the mRNA expression and protein expression were dramatically decreased 1 day after the addition of Sirt1 siRNA (Figure 3A and 3B). No change of PAI-1 expression was observed within the first two days of Sirt1 siRNA (data not shown). However, the mRNA expression and protein expression of PAI-1 were increased 3 days after the addition of the Sirt1 siRNA (Figure 3C and 3D).

In the meantime, SA-β-gal staining was performed in order to confirm the senescent phenotype. In accordance with PAI-1 expression, the number of SA-β-gal–positive cells was significantly increased 3 days after Sirt1 inhibition (Figure 3E).

According to the above-mentioned results, Sirt1 inhibition induced a premature senescent-like phenotype in the HUVECs 3 days after the addition of the Sirt1 siRNA. As such, we chose this experimental model for the next investigation.

### Rb1 did not inhibit a Sirt1 depletion-induced senescence

**Figure 4:**
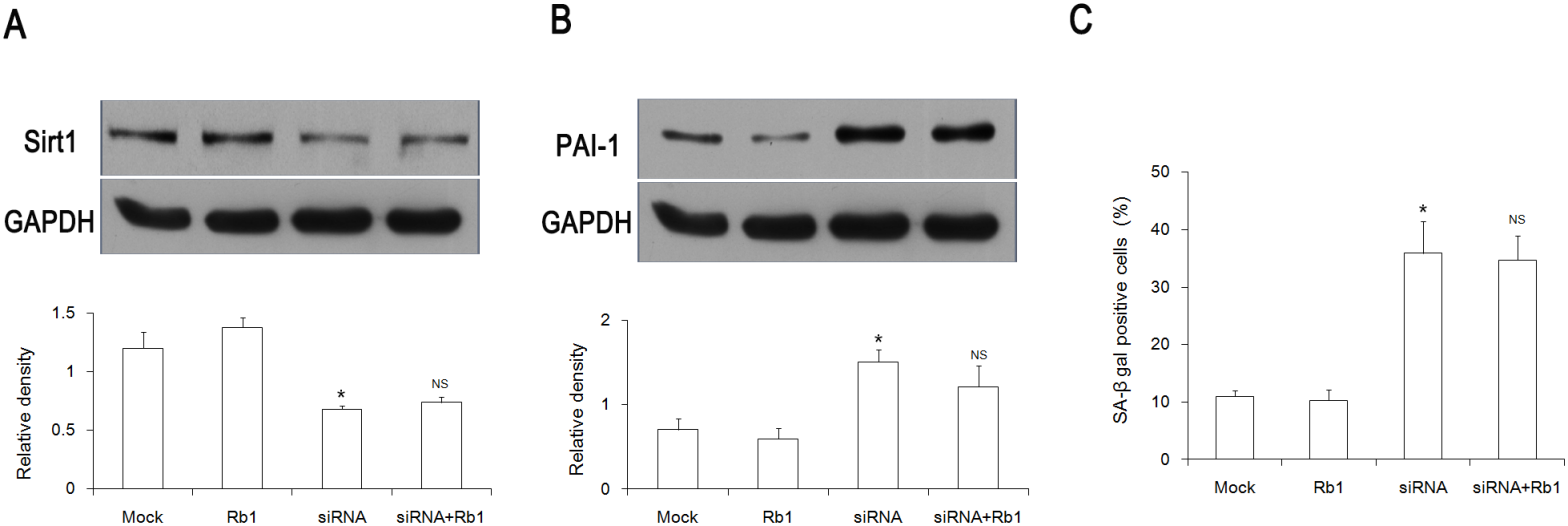
Rb1 does not inhibit Sirt1 depletion-induced senescence. (A) The Sirt1 protein level was evaluated by a Western blot analysis 72 hours after siRNA transfection with or without Rb1 supplementation. (B) The PAI-1 protein expression was examined 72 hours after siRNA transfection. (C) A total of 400 cells were counted in order to calculate the ratio of SA-β-gal–positive cells 72 hours after siRNA transfection. *P<0.01 vs Mock; NS, *P>0.05 vs* siRNA; n = 3. Mock, transfection reagent only group; Rb1, 20 µmol/L Rb1-treated group; siRNA, Sirt1 siRNA group; siRNA+Rb1, Sirt1 siRNA plus 20 µmol/L Rb1-treated group.

Our studies have shown that Rb1 can significantly prevent H_2_O_2_-induced endothelial senescence [11]. However, it is still unknown whether Rb1 has the same effect on senescence induced by Sirt1 inhibition. Compared with the siRNA group, pretreatment with Rb1 did not affect the protein expression of Sirt1 and PAI-1 3 days after Sirt1 depletion (Figure 4A, 4B). In addition, no apparent difference was seen in SA-β-gal activity with or without Rb1 supplementation (Figure 4C).

### Inhibition of Sirt1 ceased the protective effect of Rb1 against HUVEC senescence

**Figure 5:**
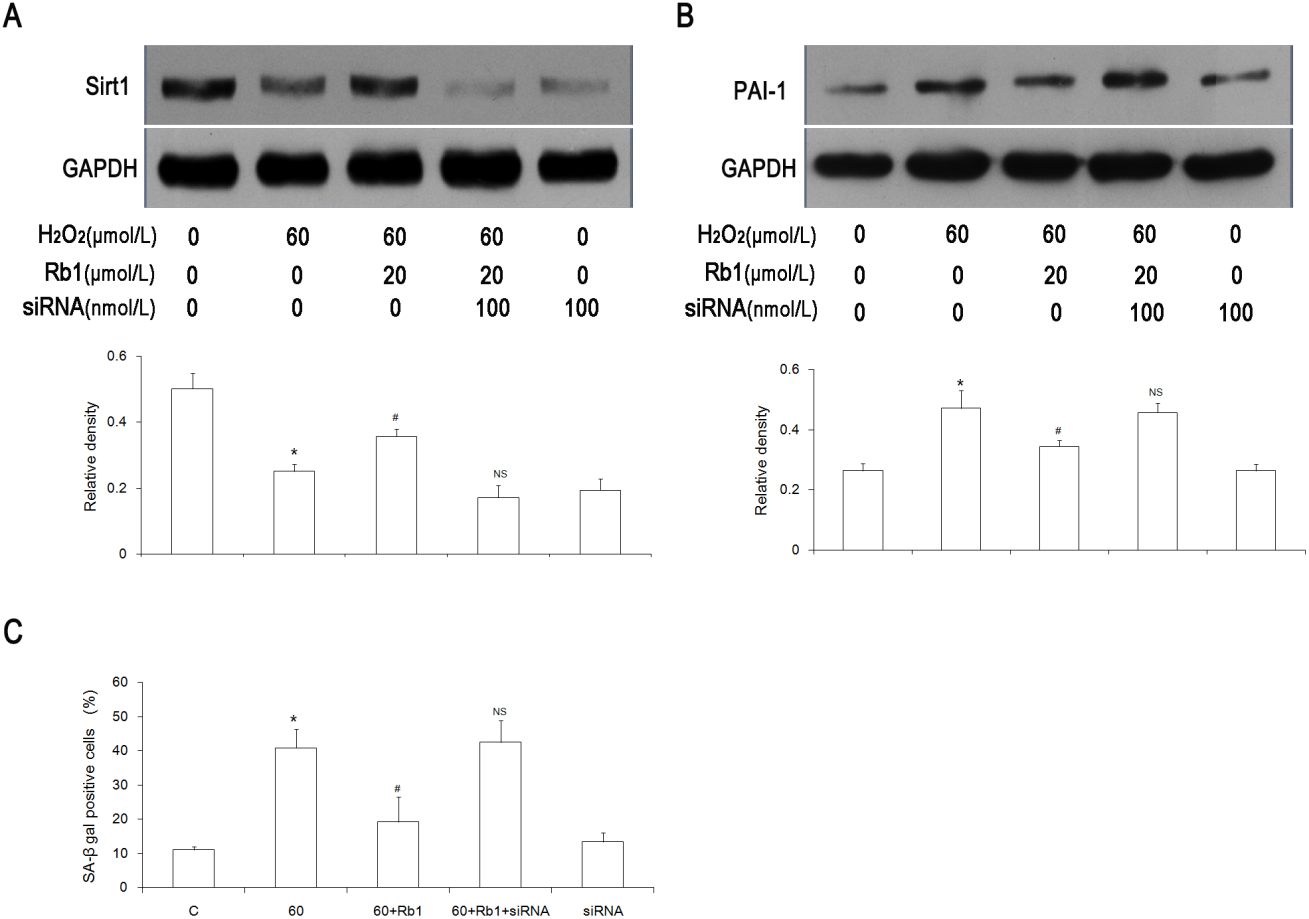
Depletion of Sirt1 ceases the effect of Rb1 against premature senescence as shown by the expression of Sirt1 (A), the expression of PAI-1 (B) and the SA-β gal staining (C). *P<0.05 vs C, *#P<0.05 vs* 60, NS, *P>0.05 vs* 60, n = 3. C, control group; 60, 60 µmol/L H_2_O_2_–treated group; 60+Rb1, 60 µmol/L H_2_O_2_ plus 20 µmol/L Rb1-treated group; 60+Rb1+siRNA, 60 µmol/L H_2_O_2_ plus 20 µmol/L Rb1 plus Sirt1 siRNA-treated group; siRNA, Sirt1 siRNA group.

Rb1 could prevent H_2_O_2_-induced endothelial senescence, but it did not affect the senescence induced by Sirt1 inhibition. In order to understand the role of Sirt1 in the prevention of Rb1 senescence, we performed the following experiments. From Figure 3, we knew that Sirt1 depletion was able to induce senescence if its depletion lasted for more than 3 days. In order to exclude this effect, the following experiments were all performed in less than 3 days. Sirt1 was inhibited with Sirt1 siRNA for 6 h, and then an H_2_O_2_-induced senescence was established as mentioned above. While Rb1-treatment did increase Sirt1 level (Figure 5A) and reduce PAI-1 (Figure 5B), the knockdown of Sirt1 prevented Rb1-mediated reduction of PAI-1 (Figure 5B). The SA-β-gal staining result also demonstrated that no improvement was achieved by Rb1 in senescent cells with Sirt1 depletion (Figure 5C).

### Rb1 decreases eNOS acetylation and promotes NO production

**Figure 6:**
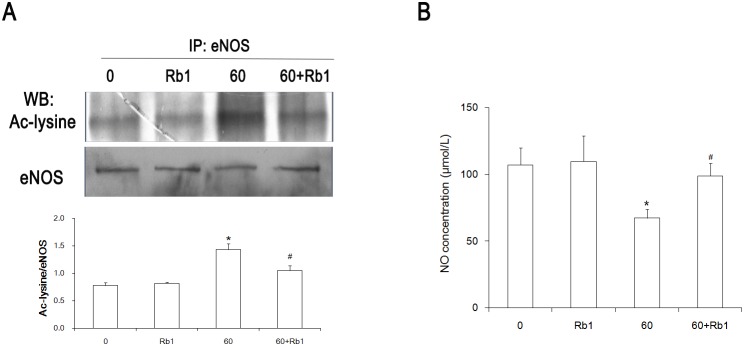
The acetylation of eNOS and production of NO are improved by Rb1 24 hours after a 1-hour H_2_O_2_ treatment. (A) A representative immunoprecipitation blot in order to evaluate the acetylation of eNOS. (B) The NO production in the cell culture supernatant was measured by using an NO assay kit. *P<0.05 vs 0, #*P<0.05 vs* 60 alone, n = 3. 0, control group; Rb1, 20 µmol/L Rb1-treated group; 60, 60 µmol/L H_2_O_2_–treated group; 60+Rb1, 60 µmol/L H_2_O_2_ plus 20 µmol/L Rb1-treated group.

From the above-mentioned results, Rb1 exhibited its protective effect on H_2_O_2_-induced senescence through the upregulation of Sirt1. Evidence has demonstrated that the acetylation of eNOS would cause endothelial dysfunction because of NO reduction [16], [25]. Sirt1 is a deacetylase and can deacetylate proteins in order to regulate cellular function. As Sirt1 was decreased in senescent HUVECs and was partially recovered with an Rb1 treatment, we were interested in determining whether Rb1 affected eNOS acetylation, so we performed the following experiments. With a senescent condition, the eNOS protein was acetylated more than that of the control group. However, if the cells were pretreated with Rb1, the eNOS protein was less acetylated (Figure 6A). This trend was the opposite of that of the Sirt1 expression (Figure 2C). Meanwhile, the NO level in the cell culture medium was detected. The NO levels were decreased in the senescent cells, but an increased NO production was observed if the cells were pretreated with Rb1 (Figure 6B).

### Rb1 reverses Sirt1 expression by inhibiting its phosphrolyation

**Figure 7:**
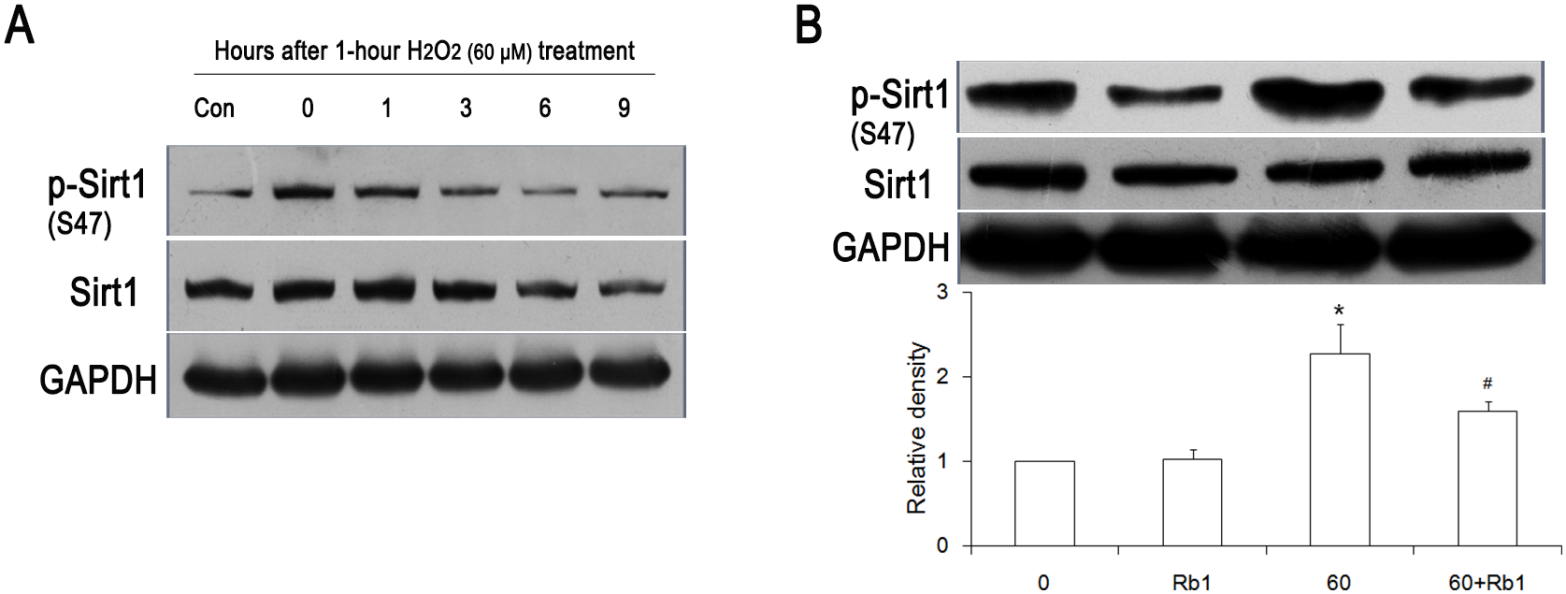
Effect of Rb1 on Sirt1 phosphorylation induced by H_2_O_2_ treatment. (A) H_2_O_2_ treatment increased the phosphorylation of Sirt1 at Ser47. (B) HUVECs were pretreated with Rb1 for 30 min and then treated with H_2_O_2_ for 1 h. *P<0.05 vs 0, #*P<0.05 vs* 60 alone, n = 3. 0, control group; Rb1, 20 µmol/L Rb1-treated group; 60, 60 µmol/L H_2_O_2_–treated group; 60+Rb1, 60 µmol/L H_2_O_2_ plus 20 µmol/L Rb1-treated group.

A recent study reveals that hyperphosphorylation of Sirt1 contributed to endothelial senescence [26]. Moreover, it has been suggested that alterations in protein level was associated with increased Sirt1 phosphorylation [16], [27]. In our study, we also found an increased phosphorylation level of Sirt1 at serine 47 during the course of senescence development (Figure 7A). However, pretreatment of HUVECs with Rb1 (20 µM) for 30 min resulted in the attenuation of Sirt1 phosphorylation compared to H_2_O_2_ treatment alone (Figure 7B).

## Discussion

In this study, we found that 60 µmol/L of H_2_O_2_ effectively induced premature senescence of the HUVECs without any apparent apoptosis. Rb1 protected the HUVECs from an H_2_O_2_-induced senescence through the stimulation of Sirt1 pathway. An increased Sirt1 expression decreased the acylation of eNOS in senescent HUVECs in order to produce more NO.

In both animal and human experiments, vascular oxidative stress develops with age [28]–[31]. During in vitro experiments, oxidative stressors such as H_2_O_2_ and ox-LDL can drive cell senescence [32]–[34]. As a disease model induced in primary cells is more clinically and physiologically relevant to human disease, the primary HUVECs are ideal for use in studying endothelium-related diseases. Hence, we chose a premature H_2_O_2_-induced senescence of HUVECs to perform our experiments.

As for the senescence model, 30–100 µmol/L of H_2_O_2_ was reported to be efficient in inducing senescence in HUVECs [11], [35], [36]. This is in agreement with our results. However, if 80 or 100 µmol/L of H_2_O_2_ was used, there were few cells left, along with an apoptotic trend. In order to exclude the effect of apoptosis in our results, we established a senescence model with 60 µmol/L of H_2_O_2._

SIRT1 has been increasingly recognized in playing a critical role in cellular senescence and aging [37], [38]. As a person’s age increases, the mRNA level of Sirt1 decreases in endothelial cells obtained from a patient’s saphenous vein that was harvested during a bypass surgery [39]. In fact, it has been reported that the endothelial cells in samples of a human atherosclerotic aorta exhibited a senescent-like phenotype, along with an increased expression of PAI-1 and SA-β-gal activity [40]. Also, Sirt1 expression was decreased in human atherosclerotic plaques and vascular smooth muscle cells undergoing senescence [41]. In endothelial cells, an H_2_O_2_ treatment caused a reduction of Sirt1 protein expression [16], [36], [42], [43]. Inhibition of Sirt1 could induce a senescent phenotype in the endothelial cells [44]. These reports suggest that Sirt1 is closely related to senescence and oxidative stress.

In our previous study, we concluded that Rb1 was able to reverse endothelial senescence by modulating the redox status [11]. Arunachalam and others reported that an oxidative stress-induced reduction in the Sirt1 level was associated with its Phosphorylation [16], [27]. In accordance with these findings, in the present study, we also found that Rb1 decreased the phosphorylation level of Sirt1 at serine 47 site which contributed much to the degradation of Sirt1 (Figure 7B). In the end, Sirt1 expression was reversed (Figure 2D).

NO is known to be involved in preventing the progression of senescence and dysfunction in endothelial cells [45], [46]. It was reported that eNOS activity and the production of NO were diminished in senescent human endothelial cells [47]. Induction of NO production by shear stress is also decreased in senescent endothelial cells. The addition of exogenous NO could delay and decrease endothelial senescence [48]. All of these results indicate that the eNOS/NO system plays a critical role in endothelial senescence.

Researchers have postulated that the Sirt1/eNOS axis is a potential target for prevention of endothelial senescence and dysfunction [17], [46], [49]. One direct relationship between them is that Sirt1 could regulate the acetylation status of eNOS, which would affect the activity of eNOS and eventually the production of NO. With an oxidative condition, the acetylation of eNOS could induce endothelial dysfunction [16]. Furthermore, our results demonstrated an Rb1 pretreatment decreased the acylation of eNOS. In the meantime, NO production was increased.

In summary, we showed that Rb1 inhibited premature, oxidative stress-induced senescence and that the enhancement of Sirt1 expression played an important role in the inhibition of HUVEC senescence. Our results suggest that Rb1-induced NO production has a protective effect against endothelial senescence.
